# Knowledge of pelvic organ prolapse in patients and their information-seeking preferences: comparing Vienna and Moscow

**DOI:** 10.1007/s00192-016-3018-4

**Published:** 2016-04-26

**Authors:** Polina Lyatoshinskaya, D. Gumina, A. Popov, M. Koch, M. Hagmann, W. Umek

**Affiliations:** 1 Department of Obstetrics and Gynecology, Medical University of Vienna, Waehringer Guertel 18-20, A-1090 Vienna, Austria; 2 Pelvic Floor Centre, Moscow Regional Research Institute of Obstetrics and Gynecology, Moscow, Russian Federation; 3 Karl-Landsteiner-Institute of Special Gynecology and Obstetrics, Vienna, Austria; 4 Department of Statistics, Medical University of Vienna, Vienna, Austria

**Keywords:** Information-seeking behaviour, Knowledge of POP, Pelvic organ prolapse, Validation of the questionnaire

## Abstract

**Introduction and hypothesis:**

We hypothesized that knowledge of pelvic organ prolapse (POP) and patient information-seeking preferences are the same in the two capital cities.

**Methods:**

First-visit patients were recruited at tertiary referral urogynaecological units in Vienna (137) and in Moscow (112). A 16-item scale was used to assess the patient knowledge of POP. The 16 items comprised 12 specific items taken from the Prolapse and Incontinence Knowledge Questionnaire (PIKQ) and four added items. The preliminary psychometric assessment of the knowledge scales in German and Russian was performed in the Vienna and in Moscow centres.

**Results:**

The mean total knowledge scores in patients in Vienna and in Moscow were not significantly different: 9.7 ± 3.5 vs. 9.8 ± 2.9 (*p* = 0.92). Patients in Vienna were more likely to answer questions about the pathogenesis of POP correctly. Patients in Moscow achieved higher scores for items assessing knowledge about the diagnosis of POP. Women in the two study groups equally preferred to obtain information about POP from medical specialists (72 % and 82 %; *p* = 0.61), followed by friends and family for patients in Vienna (25 %), and the internet for patients in Moscow (23 %). Patients in Vienna were more likely to use printed sources (18 % and 7 %; *p* = 0.001) than patients in Moscow.

**Conclusions:**

The mean level of knowledge of POP did not differ between patients in Vienna and patients in Moscow. The differences between the specific knowledge domains might be explained by different cultural preferences for seeking health information and by the range of the information sources available.

## Introduction

Considering worldwide multicultural integration, research on health information-seeking preferences and knowledge of pelvic organ prolapse (POP) in patients of different ethnic backgrounds is of increasing interest [1–7]. Lack of knowledge of pelvic floor disorders can make patients reluctant to seek professional care so that they remain dissatisfied [8, 9]. Patients may not discuss their problems with a physician because of a lack of understanding and a belief that pelvic floor disorders are a normal consequence of age [10]. Berzuk and Shay found that a low level of knowledge of pelvic floor disorders is associated with a high prevalence of pelvic floor dysfunction, and that health education is associated with an increase in quality of life and a decrease in pelvic floor symptoms [11]. Some authors have suggested that the level of knowledge and patient perception of POP are important predictors of treatment-seeking or nonseeking behaviour [5, 9, 12–14]. The importance of health education among women with POP has been highlighted in many studies [1, 5, 8, 9, 15].

The Prolapse and Incontinence Knowledge Questionnaire (PIKQ) for assessing patient knowledge of urinary incontinence and POP was designed and validated for English-speaking patients by Sha et al. [16]. This questionnaire has been used to evaluate knowledge of POP in some US and Canadian English-speaking populations [1, 5, 9, 11, 17]. A literature search yielded no studies providing information about knowledge of POP among German-speaking and Russian-speaking populations, and there are no validated versions of the PIKQ in the German and Russian languages. Extension of the research on this issue in Russia-speaking and German-speaking countries could help avoid patient misconceptions about POP in multilingual populations and help standardize the evaluation of POP knowledge in a multicultural milieu.

The aim of this study was to evaluate the level of knowledge of POP and analyse health information-seeking preferences in patients presenting at tertiary referral centres in Vienna and in Moscow. The null hypothesis was that knowledge of POP and patient information-seeking preferences are the same in Vienna and Moscow.

## Materials and Methods

### Patients and Institutions

The ethics committees of both study centres approved the study (IRB 1225/2014 and 9/2014). Patients were recruited from two dedicated urogynaecology outpatient clinics of academic tertiary referral centres in Vienna and Moscow (Medical University of Vienna and Pelvic Floor Center at the Regional Research Institute of Obstetrics and Gynaecology Moscow). At both institutions 100 – 150 surgical POP repairs are performed per year and the POP-Q system is used in all patients. All patients presenting for their initial visit with the referral diagnosis POP were asked to participate and were handed the questionnaires after consenting to participate, but before consultation with the examining physician. The referral diagnosis of POP was confirmed by pelvic examination. Healthcare workers and patients with professional medical background as well as patients lacking language skills were excluded from the study.

### Questionnaire

The study questionnaire included information on demography, level of education, annual household income, duration of symptoms, symptom severity, previous experience with POP and sexual activity. A four-point scoring system was used to assess the severity of the symptoms (Pelvic Floor Questionnaire [18]). The sources of information about POP used by patients prior to the first visit to the tertiary referral POP centres were studied according to the procedure described by Pakbaz et al. [14] and were available in both cities. Patients were asked whether they preferred seeking information through printed sources, internet, broadcast media or family, friends and healthcare providers.

The patients’ desire for information was estimated using two items assessed using a five-point Likert scale. One item reflected how strongly the patient felt about wanting to be informed before making a shared decision about treatment. The second item evaluated the patient’s need for additional information associated with trust in the physician (Table 1).

**Table 1:** Five-point Likert scales for evaluating information-seeking preferences

I. I need the information about the disease before my visit to the physician to feel free choosing the best treatment				
1. strongly disagree	2. disagree	3. neither agree or disagree	4. agree	5. strongly agree
II. I do not need to be informed about the disease; I trust my physician to make the treatment decision				
1. strongly disagree	2. disagree	3. neither agree or disagree	4. agree	5. strongly agree

### Structure of the knowledge of POP scale

A 17-item scale was used to assess patient knowledge of POP (Table 2). Twelve items assessing knowledge of pathogenesis, diagnosis and treatment of POP were taken from the PIKQ [16]. Five items were added to determine the patient knowledge of pathoanatomy, aetiology of the disease and treatment. Patients were also asked about surgical approaches, the use of mesh, and the option of uterus-preservation.

**Table 2:** POP knowledge questionnaire

Item	Domain	Questions		
Items from the PIKQ Pelvic Organ Prolapse Scale				
1	Pathogenesis	Pelvic organ prolapse is more common in young women than in old woman.		
		agree	disagree	don’t know
2	Pathogenesis	Giving birth many times may lead to pelvic organ prolapse.		
		agree	disagree	don’t know
3	Pathogenesis	Pelvic organ prolapse can happen at any age.		
		agree	disagree	don’t know
4	Treatment	Certain exercises can help to stop pelvic organ prolapse from getting worse.		
		agree	disagree	don’t know
5	Diagnosis	Symptoms of pelvic organ prolapse may include heaviness and/or pressure.		
		agree	disagree	don’t know
6	Diagnosis	A good way for a doctor to diagnose pelvic organ prolapse is by examining the patient.		
		agree	disagree	don’t know
7	Treatment	Once a patient has pelvic organ prolapse, not much can be done to help her		
		agree	disagree	don’t know
8	Pathogenesis	Heavy lifting on a daily basis can lead to pelvic organ prolapse.		
		agree	disagree	don’t know
9	Treatment	Surgery is possible treatment for pelvic organ prolapse.		
		agree	disagree	don’t know
10	Diagnosis	Doctors can run a blood test to diagnose pelvic organ prolapse.		
		agree	disagree	don’t know
11	Treatment	A rubber ring called a pessary can be used to treat symptoms of pelvic organ prolapse		
		agree	disagree	don’t know
12	Pathogenesis	People who are obese are likely to get pelvic organ prolapse.		
		agree	disagree	don’t know
Additional items in German and Russian versions of the scale				
13	Pathogenesis	Infections of the urogenital tract can cause pelvic organ prolapse.		
		agree	disagree	don’t know
14	Diagnosis	Pelvic organ prolapse is the bulging of the uterus, vagina, bladder or rectum.		
		agree	disagree	don’t know
15	Treatment	The surgical correction of pelvic organ prolapse can be done using a vaginal or abdominal method.		
		agree	disagree	don’t know
16	Treatment	The removal of the uterus is the only possible correction of pelvic organ prolapse.		
		agree	disagree	don’t know
17	Treatment	Mesh implants are used to correct pelvic organ prolapse.		
		agree	disagree	don’t know

The entire questionnaire was translated into German and Russian and re-translated into English. It was tested in 20 healthy women without any symptoms of POP to determine whether the questions were understandable and the language was appropriate.

The psychometric assessment of knowledge scales included analysis of reliability with internal consistency using the Cronbach’s alpha coefficient of each scale. A confirmatory factor analysis (CFA) was performed to evaluate the constructed validity of each scale. Absolute fit indices from the chi-squared statistic, indices from the parsimony class including the root mean square error of approximation, and comparative fit characteristics including the comparative fit index and non-normed fit index [19] were calculated. Ordinal logistic regression was applied to identify the contribution of demographic and clinical variables and information-seeking preferences to the knowledge score. Statistical analysis was carried out using software statistical environment R i386 version 3.1.2 (The R Foundation for Statistical Computing).

## Results

The questionnaire was administered to 137 POP patients in Vienna and to 112 POP patients in Moscow. Of these patients, 13 in Vienna and 7 in Moscow were excluded after completing the questionnaire because of a medical background (doctors, nurses and other healthcare providers). Six patients in Vienna were excluded because of insufficient German language skill. The plausibility-analysis yielded eight questionnaires in Vienna and ten questionnaires in Moscow with implausible answers. These questionnaires were also excluded from further analysis. Patient demographics are presented in Table 3. Patients in Moscow were more likely to have a higher level of education and lower parity than patients in Vienna.

**Table 3:** Demographic characteristics and POP history in each patient group

Characteristic	Vienna (*n* = 110)	Moscow (*n* = 95)	*p* value
Age (years), mean ± SD	59.7 ± 13.1	61.4 ± 9.0	0.27
Marital status, %			
Married	56	60	0.82
Single	6	1	0.19
Divorced	25	11	0.04
Widowed	15	28	0.07
Highest level of education, %			
Secondary school	56	19	0.001
Undergraduate	29	26	0.99
Graduate school	15	55	0.001
Menopausal status, %			
Premenopausal	25	14	0.15
Postmenopausal	74	86	0.19
Parity, mean ± SD	2.2 ± 1.1	1.7 ± 0.7	0.001
POP symptom severity score, mean ± SD	2.2 ± 0.9	2.2 ± 0.8	0.53
POP symptom duration (years), mean ± SD	7.5 ± 8.5	10.2 ± 9.4	0.04
Sexually active, %	39	23	0.10
Sexually inactive because of POP, %	22	27	0.99
Previous doctor visits because of POP symptoms, %			
None	20	18	0.89
One	44	38	0.99
Two or more	36	44	0.24
Previous POP treatment, %			
Conservative	41	28	0.43
Surgery	23	16	0.66
Mean household income (euros), %^a^			
<20,000	68	73	0.47
20,000 – 40 ,000	20	13	0.26
40,000 – 60,000	3	2	0.99
>60,000	3	1	0.73

^a^Response rates 86 % in Vienna and 73 % in Moscow

### Validation of the knowledge scale

Psychometric assessment of the POP knowledge scale in German was performed on 110 questionnaires from the Vienna centre and of the knowledge scale in Russian on 95 questionnaires from the Moscow centre. The results of the CFA suggested a good fit of the 17-item knowledge scale in German (chi-squared = 138.921) and a poor fit of the knowledge scale in Russian (chi-squared = 153.072; Table 4). Removal of item 13 from both the German and Russian knowledge scales improved the model fit and reliability of both scales (Table 5).Cronbach’s alpha coefficients for the modified German and Russian scales (0.782 and 0.667, respectively) confirmed acceptable internal consistency of both scales.

**Table 4:** Confirmatory factor analysis of the POP knowledge scales with the model fit indices

	German scale		Russian scale	
	17 items	16 items	17 items	16 items
Chi-squared^a^	138.921	121.156	153.072	127.554
*p* scaled^b^	0.102	0.120	0.019	0.058
Comparative fit index	0.951	0.952	0.711	0.793
Non-normed fit index	0.944	0.945	0.670	0.761
Root mean square error of approximation	0.038	0.038	0.052	0.047

^a^Values closer to zero indicate a better fit

^b^Values greater than 0.05 indicate a good fit

**Table 5:** Latent variable confirmatory factor analysis of the knowledge scales

Item no.	German scale (*n* = 110)			Russian scale (*n* = 95)		
	Standardized coefficient	Standard error	*p* value	Standardized coefficient	Standard error	*p* value
1	0.786	0.069	<0.0001	0.420	0.118	0.0004
2	0.599	0.108	<0.0001	0.444	0.117	0.0002
3	0.501	0.106	<0.0001	0.436	0.115	0.0002
4	0.595	0.106	<0.0001	0.534	0.109	<0.0001
5	0.337	0.117	0.0039	0.266	0.143	0.0630
6	0.256	0.149	0.0853	0.648	0.153	<0.0001
7	0.616	0.100	<0.0001	0.411	0.118	0.0005
8	0.422	0.131	0.0013	0.565	0.209	0.0068
9	0.420	0.117	0.0003	0.697	0.144	<0.0001
10	0.733	0.084	<0.0001	0.480	0.112	<0.0001
11	0.304	0.120	0.0110	0.352	0.128	0.0059
12	0.776	0.079	<0.0001	0.419	0.127	0.0009
13	0.649	0.091	<0.0001	0.018	0.132	0.8902
14	0.505	0.115	<0.0001	0.750	0.121	<0.0001
15	0.446	0.107	<0.0001	0.441	0.124	0.0004
16	0.775	0.081	<0.0001	0.481	0.134	0.0003
17	0.541	0.110	<0.0001	0.302	0.155	0.0512

### Analysis of the knowledge scales

The mean total knowledge scores (16-item scale) did not show statistically significant differences between the patient groups (9.7 ± 3.5 for patients in Vienna, and 9.8 ± 2.9 for patients Moscow; *p* > 0.05; Table 6).

**Table 6:** Modified 16-item POP knowledge scores in patients in Vienna and in Moscow

Domain	Knowledge score		
	Range	Vienna (mean ± SD)	Moscow (mean ± SD)
Pathogenesis	0 – 5	2.9 ± 1.5*	2.6 ± 1.2
Diagnostic	0 – 4	2.6 ± 0.9	3.0 ± 1.0*
Therapy	0 – 7	4.1 ± 1.9	4.2 ± 1.7
Total scale	0 – 16	9.7 ± 3.5	9.8 ± 2.9

*p ≤ 0.05

However, analysing the different domains of the scale separately, patients in Vienna were achieved higher scores for questions in the pathogenesis domain than patients in Moscow (mean scores 2.9 ± 1.5 vs. 2.6 ± 1.2; *p* = 0.05);, and patients in Moscow achieved higher scores for questions in the diagnostic domain than patients in Vienna (2.6 ± 0.9 vs. 3.0 ± 1.0; *p* < 0.05). Patients in Vienna were less well informed about the use of mesh in prolapse surgery than patients in Moscow (32 % vs. 80 % correct answers; *p* < 0.001; Table 2, item 17). There was no significant difference between the mean scores for questions in the therapy domain between patients in Vienna and patients in Moscow (4.1 ± 1.9 vs. 4.2 ± 1.7; *p* > 0.05).

### Information-seeking preferences and sources of information

Analysing preferences for health information seeking, patients in Vienna were less likely to need information about their condition before the clinical visit than patients in Moscow (five-point Likert scale mean scores: 2.9 ± 1.2 vs. 3.8 ± 1.3; *p* < 0.001). There was no difference between the patient groups in non-seeking behaviour associated with trust in the physician (five-point Likert scale mean scores: 3.7 ± 1.3 vs. 3.8 ± 1.2; *p* > 0.05).

**Fig. 1:**
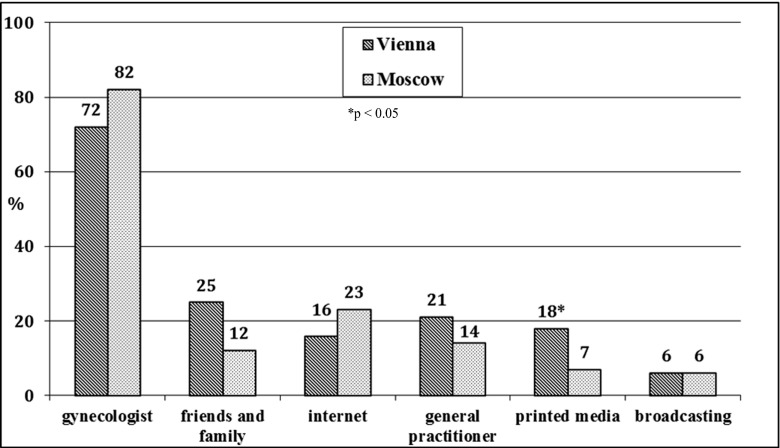
Patients’ sources of information about pelvic organ prolapse

Analysing patient preferences for sources of information, most patients in both study-groups preferred information about POP from medical specialists (72 % and 82 %, respectively; *p* > 0.05), followed by friends and family for patients in Vienna (25 %), and the internet for patients in Moscow (23 %; Fig. 1). Of the patients in Vienna and Moscow, 21 % and 14 %, respectively, were informed about POP by their general practitioner (*p* > 0.05). Patients in Vienna were more likely to use printed sources than patients in Moscow (18 % and 7 %, respectively; *p* < 0.05). Radio and television did not provide enough information in either country (6 % and 6 % respectively; *p* > 0.05).

### Predictive factors for level of knowledge

The internet and printed material as additional sources of information were the most important predictors of more knowledge of POP in both groups (Table 7).

**Table 7:** Predictive factors for higher levels of knowledge

Factor	Vienna centre (*n* = 110)		Moscow centre (*n* = 95)	
	Odds ratio	95 % confidence interval	Odds ratio	95 % confidence interval
Age	0.99	0.96 – 1.01	0.99	0.95 – 1.03
Marital status	0.49	0.17 – 1.35	0.74	0.32 – 1.71
Level of education				
Secondary school	0.55	0.27 – 1.09	0.29	0.10 – 0.77
Undergraduate	2.00	0.95 – 4.29	1.23	0.52 – 2.89
Graduate school	1.10	0.40 – 4.05	1.78	0.85 – 3.78
Menopausal status	1.19	0.54 – 2.68	1.25	0.44 – 3.49
Parity	0.85	0.62 – 1.17	1.08	0.62 – 1.89
Sexually active/inactive	1.83	0.92 – 3.67	2.65	1.10 – 6.49
Severity of POP symptoms	1.24	0.86 – 1.80	0.88	0.56 – 1.38
Previous consulting visits	1.39	0.89 – 2.21	1.01	0.63 – 1.64
Previous POP treatment	1.44	0.73 – 2.86	1.44	0.69 – 3.03
Duration of POP symptoms	1.01	0.96 – 1.05	0.98	0.95 – 1.02
Household income	1.62	0.95 – 2.83	1.49	0.77 – 2.98
Stronger information-seeking preferences	1.36	1.03 – 1.82	1.08	0.80 – 1.46
Patient’s trust in physician	0.69	0.52 – 0.89	0.70	0.51 – 0.90
Sources of information about POP				
Family and friends	2.31	1.07 – 5.07	1.59	0.48 – 5.43
General practitioner	1.25	0.57 – 2.76	0.21	0.06 – 0.65
Gynaecologist	1.72	0.79 – 3.78	2.89	1.07 – 8.28
Newspapers and magazines	4.25	1.69 – 11.18	6.56	3.05 – 24.44
Radio and television	4.05	0.92 – 21.31	1.42	0.28 – 7.32
Internet	4.67	1.77 – 11.42	5.12	2.06 – 13.26

Odds ratios and 95 % confidence intervals were extracted from the ordinal logistic regression model

If the confidence interval contains the relative risk of 1.00, the factor is not significantly predictive of the knowledge score; if the confidence interval is less than 1.00, the factor is significantly predictive of a decreases in score, and if more than 1.00 is significantly predictive of an increase in the score

A higher information-seeking preference score was a significant predictor of a higher knowledge score in patients in Vienna, and a lower trust in physician score was a significant predictor of a higher knowledge score in patients in Vienna and patients in Moscow (OR 0.69, 95 % CI 0.52 – 0.89, and OR 0.7, 95 % CI 0.51 – 0.9, respectively). Additional predictors of the knowledge score in patients in Moscow were a low level of education (OR 0.29, 95 % CI 0.1 – 0.77) and a sexually active patient (OR 2.65; 95 % CI 1.1 – 6.49).

## Discussion

This study focused on the evaluation of patient knowledge of POP at tertiary prolapse centres in Vienna and Moscow, and confirmed the hypothesis that levels of knowledge of POP do not differ between these two cities. To evaluate patient knowledge of POP in German and Russian populations we developed validated and reliable extended versions of the POP Knowledge Scale in the German and Russian languages. Translation and psychometric adaptation of reliable questionnaires for patients speaking different languages allows research to be extended in other countries [7, 18, 20–22].

We did not find any significant difference in the mean total knowledge score in women with POP between Vienna and Moscow (9.7 vs. 9.8). Sha et al. found a mean knowledge score of 8.2 using the original 12-item POP scale [16]. These authors also recruited patients presenting at tertiary care POP centre. Lower average PIKQ scores were found by Mandimika et al. [9] in English-speaking community-dwelling women (5.5) and by Dunivan et al. [1] in elder American Indian women (5.4). The percentile rank should be calculated to compare the mean scores of 16-item and 12-item POP knowledge scales.

The analysis comparing knowledge domains did not reveal any significant differences between the mean scores of the patients in Vienna and Moscow. However, the detailed analysis of the therapy items revealed that the patients in Moscow were more informed about the use of mesh for prolapse correction than patients in Vienna (80 % vs. 32 % correct answers). This may be explained by the fact that mesh implants are more commonly used in Russia to correct prolapse [23], in contrast to the current practice in Austria [24]. In a study of patient knowledge of vaginal mesh surgery, Brown et al. found that nearly two thirds of patients presenting at a tertiary referral centre in Michigan had received information about this issue from medical professionals or from the television [25].

Level of knowledge is an integrative parameter that may depend on numerous social and demographic variables. One of the most important predictive factors is the patient’s wish to be informed. It has been suggested that not all patients want to be involved in medical decision making and that unnecessarily detailed information can induce emotional stress in passively inclined patients [26]. Sung et al. investigated information-seeking preferences in women with pelvic floor disorders using the Autonomy Preference Index (API) [27]. Given that there were no validated versions of this scale in either German or Russian, we tried to formulate our questions taking into consideration the cultural particularities of the doctor–patient relationships and trust in physicians in the two countries. The patients in Moscow showed significantly stronger information-seeking preferences prior to the clinical visit in contrast to patients in Vienna. This may be explained by differences between the “models” of doctor–patient relationships between the two populations [28]. While an “informative” model of the doctor–patient relationship is more popular in German-speaking areas, a more “paternalistic” character of the interaction predominates in Russian culture historically.

The higher level of knowledge of POP was associated with stronger preferences for information seeking in patients in Vienna, but not with greater trust in the physician. In contrast, the trust in physician predicted the lower scores of the knowledge scale in patients in both Vienna and Moscow. Similar findings have not been reported previously. In contrast to the findings of Mandimika et al. [9], we could not find any association between knowledge scores and parameters including age, parity and household income in both groups. It is possible that there was some inaccuracy in the income analysis due to the low income variability and the poor response rate to this item in this study. This is similar to the finding of Pakbaz et al. [14] who found that POP patients in Vienna and Moscow prefer to obtain their information from specialized health providers. Given that the women rated the physician’s opinion as one of the most important factors in making a decision, physicians should avoid the authoritative form of the patient–physician interaction and provide the patient with all relevant information to make a shared decision about therapy. We believe that providing more information about new diagnostic tools and prolapse therapeutic procedures in press media sources or via the Internet could significantly improve the understanding of POP in patients in Vienna and Moscow.

In summary, our findings did not suggest global knowledge gaps in patients with prolapse in Vienna and Moscow. The differences in specific knowledge might be explained by the different cultural preferences for seeking health information and by the range of information sources available. The authors would like to encourage further research in both populations to investigate more fully the sociocultural reasons for patients’ misconceptions about POP.
